# DNA methylation and lipid metabolism are involved in GA-induced maize aleurone layers PCD as revealed by transcriptome analysis

**DOI:** 10.1186/s12870-023-04565-5

**Published:** 2023-11-22

**Authors:** Yequn Wu, Jiaqi Hou, Ruifei Ren, Zhenfei Chen, Mengxia Yue, Le Li, Haoli Hou, Xueke Zheng, Lijia Li

**Affiliations:** 1 State Key Laboratory of Hybrid Rice, College of Life Sciences, Wuhan University, Wuhan, 430072 China; 2 College of Food, Xinyang Agriculture and Forestry University, Xinyang, 464000 China

**Keywords:** DNA methylation, HDACs, H_2_O_2_, Lipid metabolism, Maize aleurones, Programmed cell death (PCD)

## Abstract

**Background:**

The aleurone layer is a part of many plant seeds, and during seed germination, aleurone cells undergo PCD, which is promoted by GA from the embryo. However, the numerous components of the GA signaling pathway that mediate PCD of the aleurone layers remain to be identified. Few genes and transcriptomes have been studied thus far in aleurone layers to improve our understanding of how PCD occurs and how the regulatory mechanism functions during PCD. Our previous studies have shown that histone deacetylases (HDACs) are required in GA-induced PCD of aleurone layer. To further explore the molecular mechanisms by which epigenetic modifications regulate aleurone PCD, we performed a global comparative transcriptome analysis of embryoless aleurones treated with GA or histone acetylase (HAT) inhibitors.

**Results:**

In this study, a total of 7,919 differentially expressed genes (DEGs) were analyzed, 2,554 DEGs of which were found to be common under two treatments. These identified DEGs were involved in various biological processes, including DNA methylation, lipid metabolism and ROS signaling. Further investigations revealed that inhibition of DNA methyltransferases prevented aleurone PCD, suggesting that active DNA methylation plays a role in regulating aleurone PCD. GA or HAT inhibitor induced lipoxygenase gene expression, leading to lipid degradation, but this process was not affected by DNA methylation. However, DNA methylation inhibitor could regulate ROS-related gene expression and inhibit GA-induced production of hydrogen peroxide (H_2_O_2_).

**Conclusion:**

Overall, linking of lipoxygenase, DNA methylation, and H_2_O_2_ may indicate that GA-induced higher HDAC activity in aleurones causes breakdown of lipids via regulating lipoxygenase gene expression, and increased DNA methylation positively mediates H_2_O_2_ production; thus, DNA methylation and lipid metabolism pathways may represent an important and complex signaling network in maize aleurone PCD.

**Supplementary Information:**

The online version contains supplementary material available at 10.1186/s12870-023-04565-5.

## Background

Programmed cell death (PCD) is associated with specific physiological, biochemical, and cellular structural changes, including an increase in reactive oxygen species (ROS) and chromatin condensation [1, 2]. This is a fundamental mechanism for regulating several aspects of plant development and stress response. These changes are often driven by plant hormones, developmental factors, and external biotic and abiotic stress [3, 4]. The aleurone layer is a component of seeds of many plant species, and during seed germination, aleurone cells undergo PCD, which is promoted by gibberellin (GA) from the embryo [5]. In the GA signaling pathway, GA is perceived by and binds to the plasma membrane receptor GID1, which then interacts with the DELLA repression protein. This complex results in degradation of the DELLA protein through the E3 ubiquitin ligase, which can activate a downstream myb-like transcription factor (GAMyb). This factor regulates α-amylase gene transcription in the aleurone layers [6]. GA can also lead to the production of ROS, especially hydrogen peroxide (H_2_O_2_), which plays an important role in regulating PCD [7]. In addition to ROS and GAMyb, histone acetylation and protein phosphorylation have been identified as major PCD regulators in the aleurone cell death process [8, 9]. However, the numerous components of the GA signaling pathway mediated aleurone PCD remain to be identified.

Eukaryotic chromatin is composed of histones and DNA. The histone is subjected to different modifications at its N-terminal tail including acetylation regulated by histone acetyltransferases (HATs) and histone deacetylases (HDACs) [10, 11], and DNA can be methylated at cytosine by DNA methyltransferase. Histone acetylation is well characterized, which is always associated with active gene expression. By contrast, histone deacetylation of the genome often leads to transcriptional silencing [12]. Cytosines in CG, CHG and CHH DNA sequence contexts can be methylated by the DNA methyltransferases and DNA methylation can be removed through DNA demethylases. A number of studies have indicated that the DNA methylation is always correlated to compact chromatin and decreases accessibility of transcription factors to genes, whereas DNA demethylation leads to decondensed chromatin, which unsilences gene expression [13, 14]. However, recent studies have also showed a broad inhibitory role of active DNA demethylation in gene regulation in plants [15]. Several pieces of evidence reveals that a HAT complex regulates DNA demethylation by facilitating the recruiting of DNA demethylases to the target loci [16]. DNA methylation is frequently associated with histone hypoacetylation to suppress gene expression [17]. The application of the HDAC inhibitor TSA could cause selective loss of DNA methylation in Neurospora [18] and histone H4 hyperacetylation reduces DNA methylation levels [19]. It is well established that DNA methylation can lead to the recruitment of HDACs [20, 21]. As a result, DNA methylation/demethylation, which is always coupled with histone modification, influences a variety of biological processes, such as plant growth, response to stress and cell apoptosis induction through inducing specific changes in gene expression [22–24].

The aleurone layer is a model material for investigating the PCD process in plants. There is an intimate and complex regulation network involving hydrolase synthesis signaling and plant hormone signaling during this PCD process [5]. However, few genes and transcriptomes have been studied thus far in aleurone layers to improve our understanding of how PCD occurs and how the regulatory mechanism functions during PCD. In this study, we used second generation of RNA sequencing (RNA-seq) techniques to produce global transcriptome data during aleurone cell death. Through analysis of differentially expressed genes and different biological pathways, we found that genes related to DNA methylation and lipid metabolism as well as ROS signaling showed significant differences in mRNA transcription. Further studies including qRT-PCR, the contents of lipids and DNA methylation established a link between lipid metabolism, DNA methylation and ROS during GA or the HAT inhibitor induced maize aleurone layer PCD.

## Results

### Identification of target genes that are regulated by GA or the HAT inhibitor in maize aleurones

**Fig. 1:**
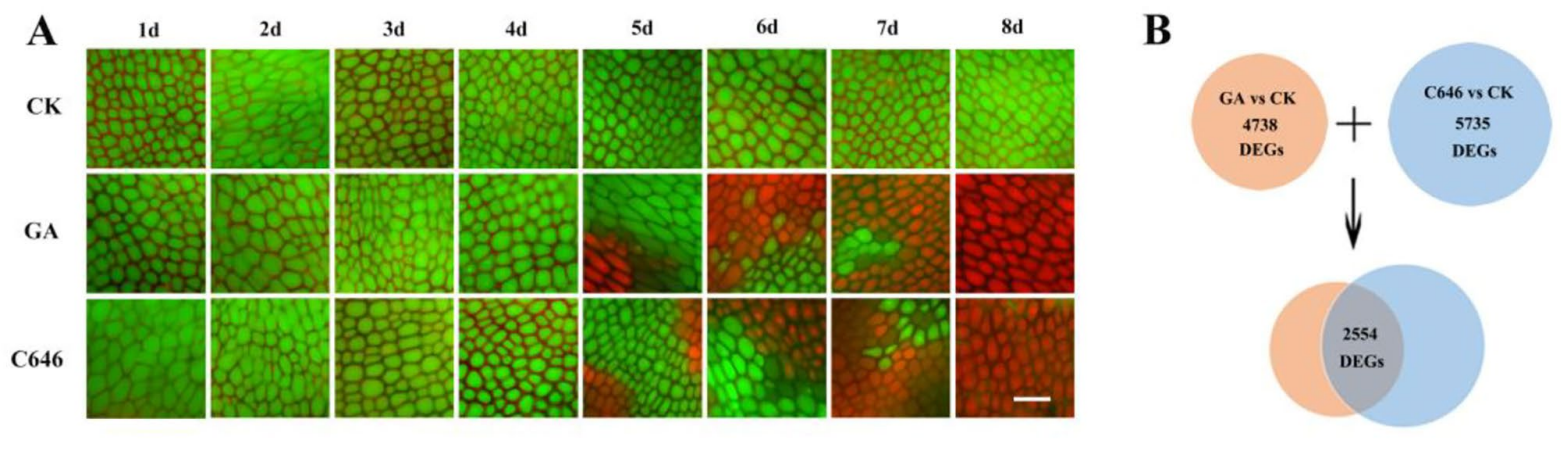
Comparison of differentially expressed genes (DEGs) between the control, GA and C646 treated aleurone layers through transcript profile analysis. (**A**) The representative images of aleurone layers after cultured with GA or C646 from 1 to 8 d were obtained after FDA/PI staining by an epifluorescence microscope. Green represents live cells, and red represents dead cells. Bar = 100 μm. (**B**) Venn diagrams showing that 2554 DEGs were common during GA or C646 induced PCD

During seed germination, embryo-produced GA promotes PCD in plant aleurone layers [25]. We previously showed that programmed cell death (PCD) occurred in maize embryoless aleurone layers after treatment with HAT inhibitors as well as GA [9]. Thus, to explore the role of epigenetic modification and signaling regulation network during GA-induced PCD in the maize aleurones, we sampled intact and maize embryoless aleurones cultured in the presence of GA or C646 (a HAT inhibitor) at the different time points (from 1 d to 5 d) (Fig. 1A), which corresponded to the key stages at which various storages are metabolized and then cells begin to be dead, and three biological replicates were applied for every sample at every time point. The untreated embryoless aleurones, which can live up to a few months and may not undergo PCD, were used for the control, and therefore, a total of 45 RNA libraries were constructed. An Illumina HiSeq Xten platform was used to sequence these libraries. Finally, we totally collected 401.11Gb Clean Data approximately (Supplemental Table S1) and Clean Reads of each sample matched the unique reference sequence 81.57%~88.77%. After careful filtering and further bioinformatic analysis, a total of 45,856 genes were collected from all 45 samples in the study. No obvious differences were observed in the number of reads among the 45 samples. Among these, 9,134 new genes were identified, 6,689 of which were functionally annotated. In total, 7,919 differentially expressed genes (DEGs) were filtered with criteria of P value < 0.05 and |log2 (foldchange)| ≥1.0 (Supplemental Table S2). After further analysis, 4,738 DEGs were identified from GA-treated aleurones while 5,735 DEGs were isolated from C646-treated aleurones; and 2,554 DEGs were found to be common under these two treatment conditions (Fig. 1B), which suggested that these DEGs were major PCD-related genes.

To explore the potential functions of 2554 DEGs (Supplemental Table S3) in response to GA or C646, we searched the non-redundant protein sequence database in NCBI by using the Blast2GO [26]. The results revealed that 1898 genes could be grouped into different categories such as 1337 DEGs involved in cellular components, 1417 DEGs participating in biological processes, and 1445 DEGs showing molecular functions. We further calculated the functional category distribution frequency based on the level 2 and the level 3 (Fig. S1). The 1417 DEGs involved in the biological process could be further grouped into 22 different types of biological processes: the 894 DEGs participated in the cellular process, the 924 DEGs were involved in the metabolic process, the 461 DEGs were associated with responses to environmental stress. These results showed that metabolic and cellular processes contained about 40% of these annotated DEGs in biological processes, suggesting that cellular activities and metabolic processes were significantly regulated by GA or C646 in the maize aleurone layers. The Kyoto Encyclopedia of Genes and Genomes (KEGG) pathway analysis of these DEGs showed that 471 functional genes were involved in 113 KEGG pathways (Fig. S2).

### GA or C646 induces PCD in maize aleurones by altering global DNA methylation of chromatin

**Fig. 2:**
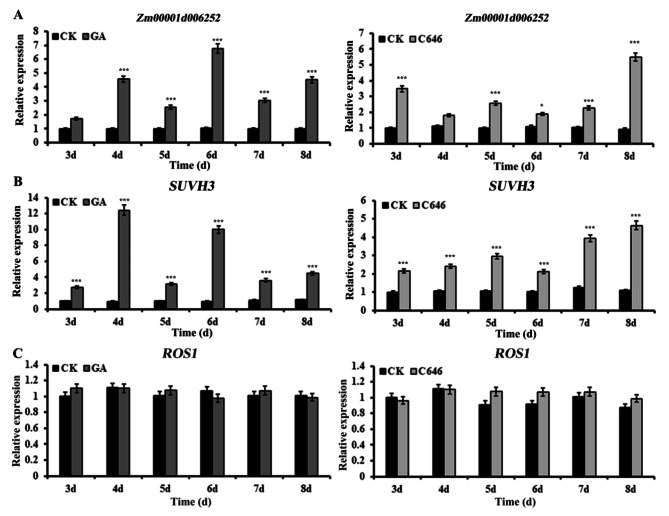
Transcript levels of DNA methylation related genes in maize aleurone layers under GA or C646 treatment revealed by qRT-PCR. (**A**) The expression of DNA methylase *Zm00001d006252*. (**B**) The expression of histone H3 methyltransferase *SUVH3*. (**C**) The expression of DNA demethylase *ROS1*. The relative expression value of the control group at 3 d was defined as 1. The data were obtained from three independent biological experiments. *, *P <* 0.05, ***, *P <* 0.01, as compared with the control with the Student’s *t* test

**Fig. 3:**
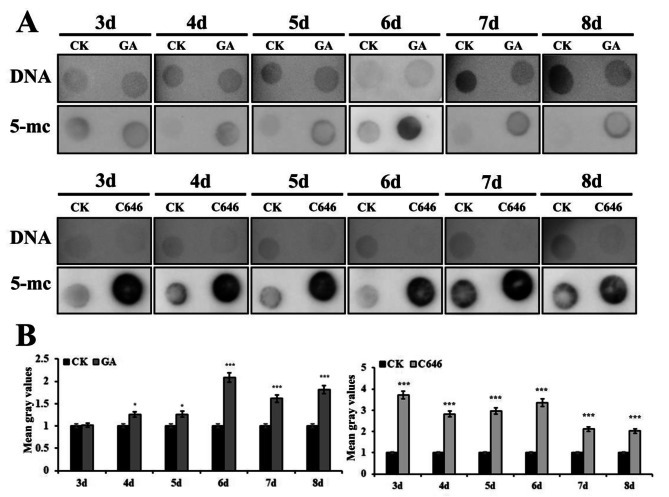
The global DNA methylation level of chromatin in aleurone layers after GA or HAT inhibitor C646 treatment. (**A**) Dot blot analysis of DNA methylation levels after treated with GA and C646. (**B**) Quantitative analysis of dot blot results was performed by ImageJ. Each assay was repeated three times for every sample in three independent experiments. *, *P <* 0.05, ***, *P <* 0.01, by Student’s *t* test

DNA methylation has been shown to play an important role in many biological processes. It has been reported that DNA methylation involved in PCD process [27]. It has been observed that several genes involved in DNA methylation were differentially expressed after exposure to GA or C646. To explore the possible relationship between DNA methylation and GA-induced aleurone layers PCD process, we focused on examining the expression of DNA methylation related genes. The qRT-PCR results showed the expression of *Zm00001d00625*, which was involved in DNA methylation, was significantly up-regulated after treatment with GA or C646 for 3 d (Fig. 2A). The expression of a histone H3 lysine 9 (H3K9) methyltransferase *SUVH3* (Zm00001d043135), which dimethylated H3K9 (H3K9me2) and facilitated the DNA methyltransferase CHROMOMETHYLASE 3 function [28, 29], was also significantly up-regulated after GA or C646 treated for 3 d (Fig. 2B). On the contrary, the expression of *ROS1* (Zm00001d053251), which functions in the first step of the DNA demethylation pathway, was not remarkably changed after treatment with GA or C646 (Fig. 2C). These results suggested that DNA methylation was strongly affected during the GA or C646 induced PCD process. To confirm the relative activity of DNA methylation/demethylation enzymes, the total DNA methylation state of chromatin of aleurone cells was detected by DNA dot-blot immunoassay with an anti-5mC antibody after GA or C646 treatment. The genome-wide DNA methylation degree was remarkably increased not only in GA-treated aleurones, but also in C646-treated aleurones compared with untreated aleurones (Fig. 3A and B).

**Fig. 4:**
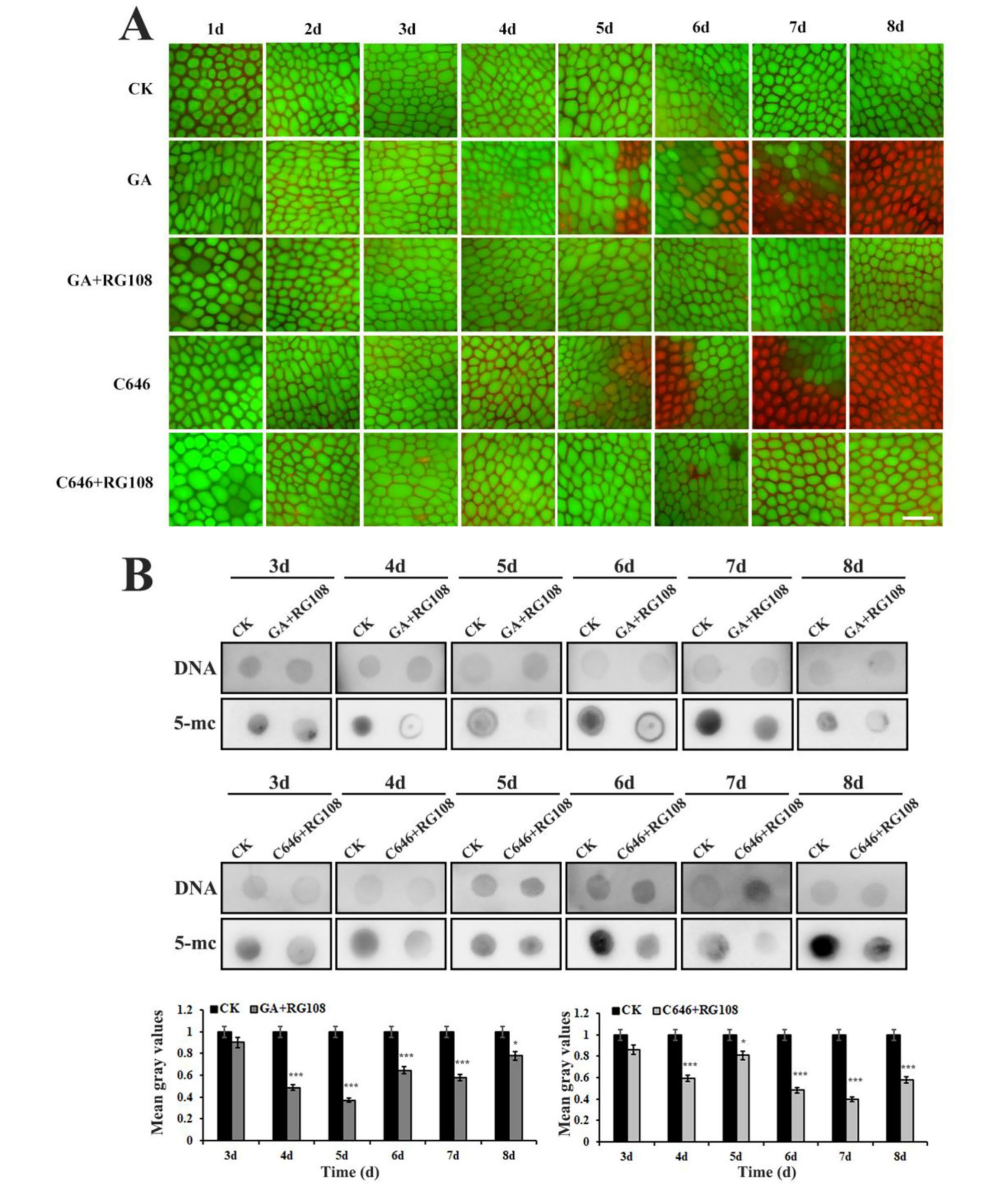
Analysis of effects of the DNA methylation inhibitor RG108 on GA or C646 mediated PCD in aleurones monitored by FDA/PI staining. (**A**) The addition of RG108 suppressed GA or C646 induced PCD in maize aleurones. Bar = 100 μm. (**B**) Dot blot analysis of DNA methylation levels after treatment with GA or C646 in the absence or presence of DNA methylation inhibitor RG108. The mean gray value for DNA methylation after treatment with RG108 was significantly reduced compared with the value of the control. Each assay was repeated three times for every sample in three independent experiments. *, *P <* 0.05, ***, *P <* 0.01, by the Student’s *t* test

The finding that the DNA methylation level was increased in aleurones after treatment with GA or C646 promotes us to hypothesize that increased DNA methylation has a positive effect on PCD in maize aleurones. Thus, we treated maize embryoless aleurone layers with the DNA methylation inhibitor RG108 in the presence of GA or C646. The cells in GA or C646-treated aleurone layers alone started to die after 5 d, and all cells died after 8 d of incubation (Fig. 4A). In contrast, almost all cells were still alive after treatment with GA or C646 in the presence of RG108 for up to 8 d (Fig. 4A). Similarly, we also performed DNA Dot blotting assay using an anti-5mC antibody. As expected, Anti-5mC signals decreased after treatment with RG108 or even with RG108 in the presence of GA or C646 as compared with GA or C646 treatment alone (Fig. 4B). To exclude the possibility that GA or C646 treatment nonspecifically inhibited PCD, we treated maize aleurones with another DNA methylation inhibitor, 5-AC, and observed the same effects (Fig. S3). These results indicated that DNA methylation is involved in the GA-mediated PCD process of maize aleurone layers.

### Changes in the lipid metabolism pathway in aleurones under GA or C646 treatment

**Fig. 5:**
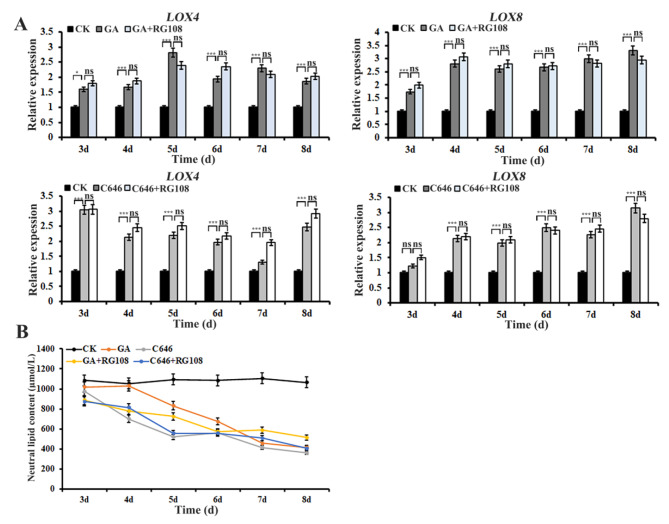
Effects of GA or C646 on lipid breakdown in maize aleurone layers in the absence or presence of RG108. (**A**) qRT-PCR was used to verify the detailed expression profiles of genes involved in lipid catabolism. (**B**) Detection of endogenous lipid contents in aleurones after treatment with GA, C646, GA plus RG108 or C646 plus RG108 as compared with the control. The x-axis indicates the different time points; the y-axis indicates relative expression values or neutral lipid contents. Each assay was repeated three times for every sample in three independent experiments. ns indicates not significant, asterisks indicate statistically significant differences. *, *P <* 0.05, ***, *P <* 0.01, as compared with the control with the Student’s *t* test

**Fig. 6:**
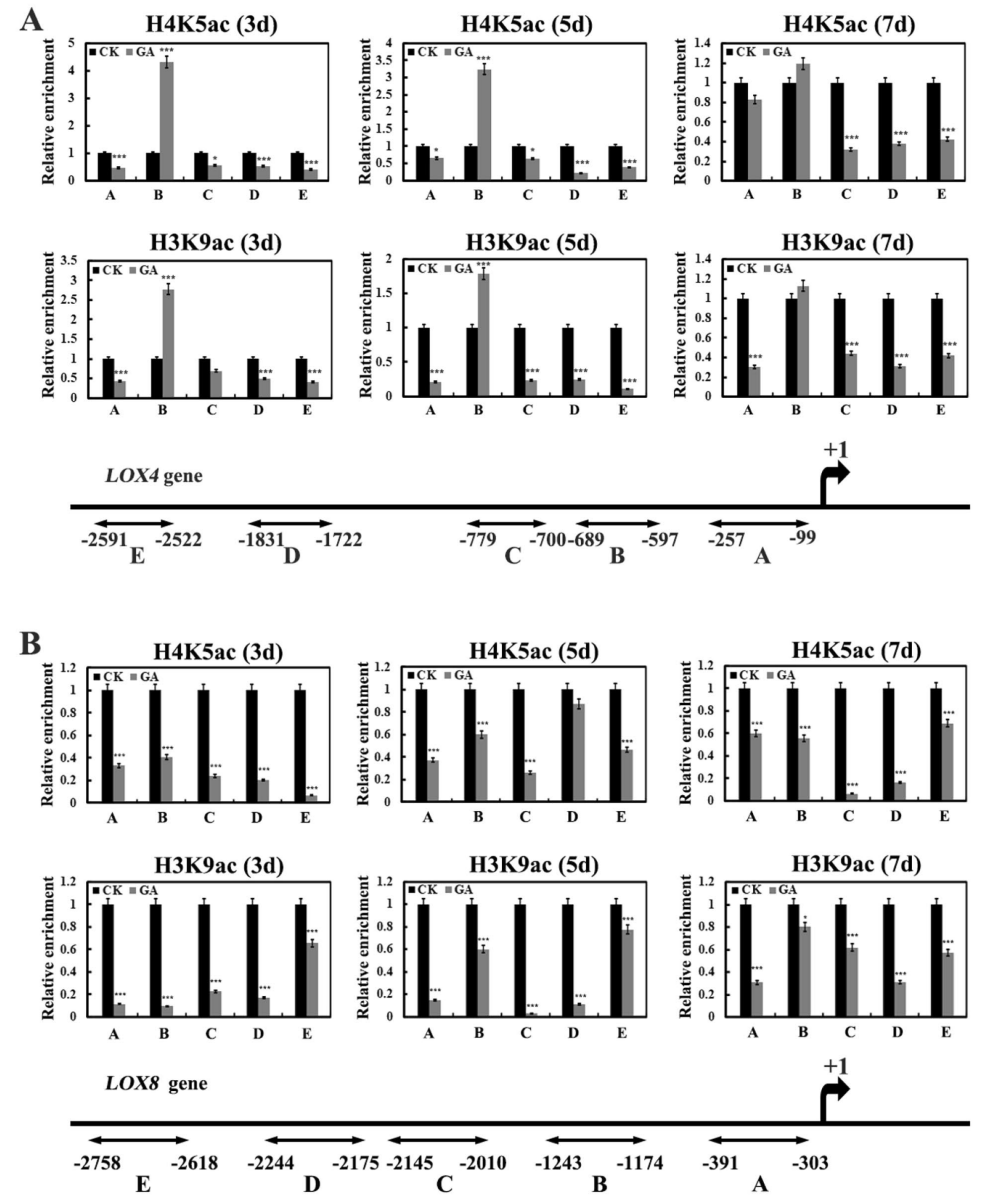
Chromatin immunoprecipitation (ChIP) results of *LOX4* and *LOX8* gene. (**A**) H3K9ac and H4K5ac levels in the promoter regions (sets A–E) of the *LOX4* gene after GA treatment in maize aleurone layers during the PCD process and schematic representation of the *LOX4* gene. (**B**) H3K9ac and H4K5ac levels in the promoter regions (sets A–E) of the *LOX8* gene after GA treatment in maize aleurone layers during the PCD process and schematic representation of the *LOX8* gene. Each assay was repeated three times for every sample in three independent experiments. *, *P <* 0.05, ***, *P <* 0.01, by Student’s *t* test

Aleurone cells store large amounts of neutral lipid in specialized oleosome [30]. Lipid metabolism includes a wide range of lipid classes, such as glyoxylate metabolism, linoleic acid metabolism and acid degradation [31]. Lipoxygenase catalyzed lipid degradation in the cereal aleurone layer during early germination [32, 33]. Changes in the expression levels of a subset of genes implicated in different lipid metabolism signaling pathways were observed and these DEGs were significantly influenced by GA or C646 (Fig. S4). The qRT-PCR results showed that the expression of two lipoxygenases, *LOX4* (Zm00001d033624) and *LOX8* (Zm00001d003533), were significantly up-regulated after treatment with GA or C646 for 3–5 d (Fig. 5A). These results indicated that GA might induce lipid degradation, which was achieved through increasing the expression levels of catabolism genes. To verify this conclusion, we analyzed the contents of total lipids in aleurone layers. The results revealed that the significant reductions in the total lipid contents were observed in aleurones when subjected to GA or C646 (Fig. 5B). To examine whether DNA methylation participated in regulation of lipid consumption, the lipid content and lipid metabolism-related genes were assessed in aleurones after treated with RG108 in the presence of GA or C646. The result showed that RG108 could not prevent GA or C646 induced up-regulation of lipid metabolism-related genes and lipid degradation (Fig. 5A and B), suggesting that lipid metabolism signaling might not be affected by DNA methylation. Next, we performed chromatin immunoprecipitation analysis of acetylation levels at the promoter of genes *LOX4* and *LOX8* in the aleurones treated with or without GA. GA induced hypoacetylation at the promoter regions A, C, D and E except increased acetylation levels at the region B in the gene *LOX4* (Fig. 6A) and at all promoter regions analyzed in the gene *LOX8* (Fig. 6B). These data are similar to the results for ROS-related genes in GA-treated maize aleurones reported previously by [9]. Therefore, we conclude that GA enhances activity of HDACs, leading to the excessive expression of lipid oxidation genes and lipid hydrolysis.

### Reactive oxygen species (ROS) signaling regulates GA or C646 induced PCD in maize aleurone layers

**Fig. 7:**
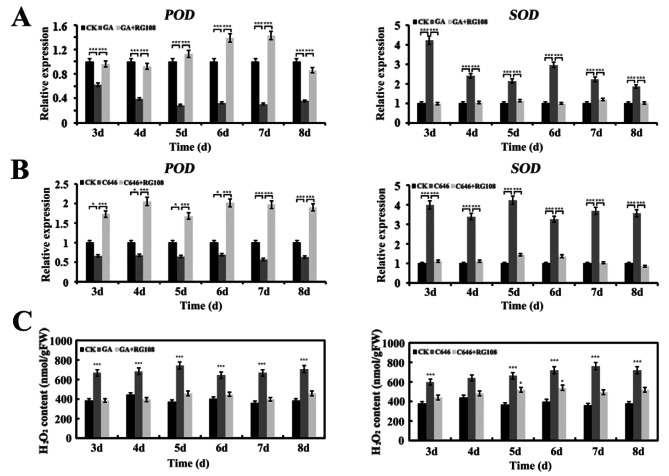
Accumulation of H_2_O_2_ in maize aleurone layer induced by GA, C646 and methylation inhibitor RG108. (**A**) Quantitative real-time PCR analysis of POD and SOD genes after GA or GA plus RG108 treatment. (**B**) Quantitative real-time PCR analysis of *POD* and *SOD* genes after C646 or C646 plus RG108 treatment. (**C**) The content of hydrogen peroxide. The methylation inhibitor RG108 inhibits the production of hydrogen peroxide. The abscissa indicates the different stages, and the ordinate indicates the relative expression level of the genes or H_2_O_2_ contents. The data obtained from three independent biological experiments. *, *P <* 0.05, ***, *P <* 0.01, as compared with the control with the Student’s *t* test

**Fig. 8:**
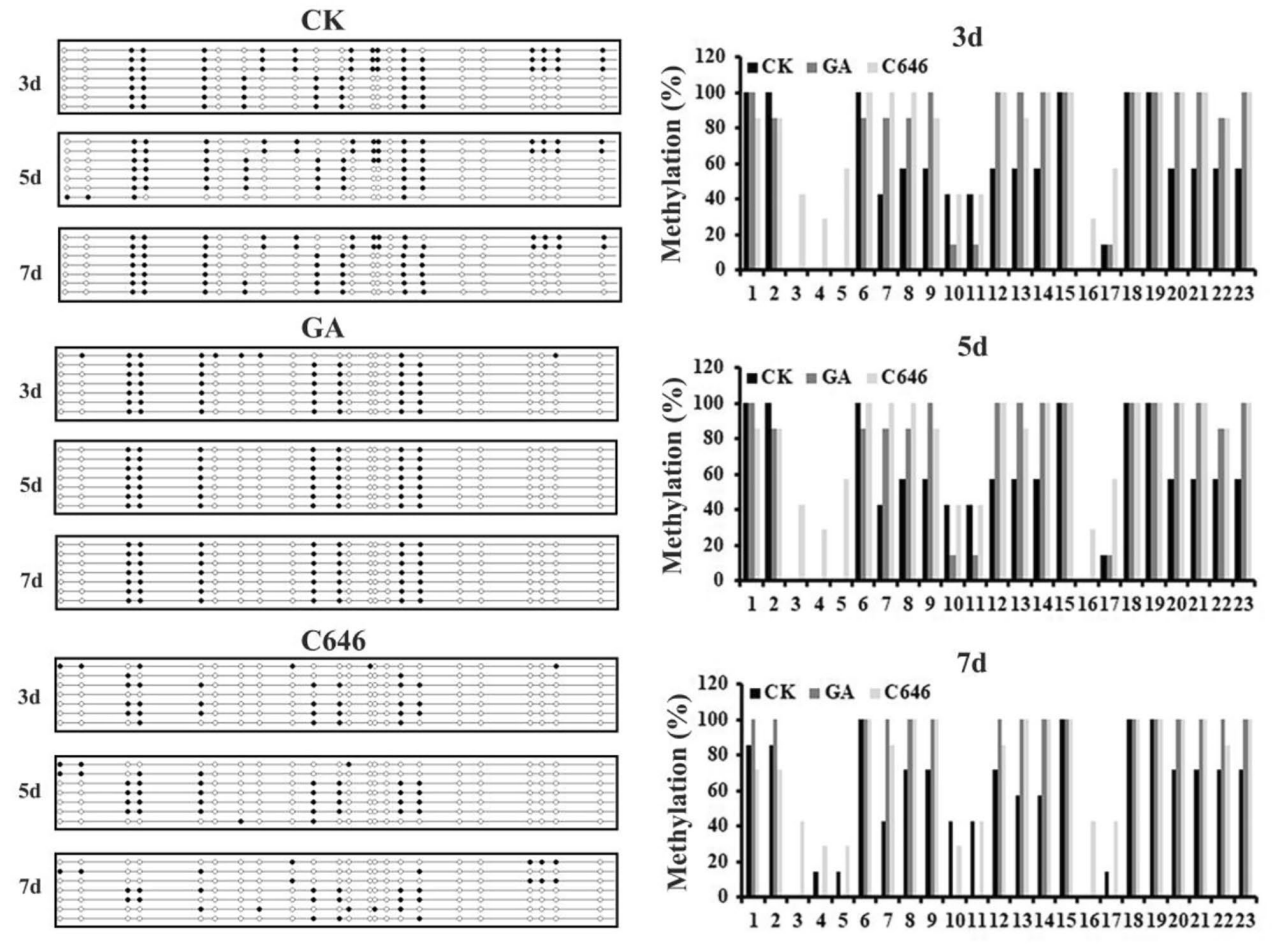
Sodium bisulfite sequencing showed the methylation status of the *SOD* gene promoter in GA or C646 treatment as compared with untreated group. The filled circle represents unmethylated CpG dinucleotides and the open circle represents methylated CpG dinucleotides. Hypermethylation of *SOD* gene promoter in aleurone layers after GA or C646 treatment as compared with untreated group showed by quantitative analysis of methylation density at each CpG.

The ROS signaling pathway plays an important role in seed germination and aleurone PCD [9, 34]. ROS includes hydrogen peroxide (H_2_O_2_), superoxide anion (O_2_^–^) and hydroxyl radical (OH^–^), and is produced as intracellular by-products of biological aerobic metabolism, mainly from lipid metabolism and sugar metabolism [35, 36]. The above-mentioned pathways might both be involved in ROS production in maize aleurone PCD. Generally, the ROS was accumulated through enhancing ROS biosynthesis and suppressing catabolism. H_2_O_2_ has been suggested to act as a key signal molecule in regulating PCD in aleurone cells [9, 37]. Therefore, we focused on characterizing genes involved in H_2_O_2_ degradation and synthesis during PCD in aleurone cells. Expression of a peroxidase (POD) gene, *POD* (Zm00001d014467), which mainly hydrolyzes H_2_O_2_, was significantly reduced whereas expression of the superoxide dismutase (SOD) (Zm00001d045384) gene, which catalyzes superoxide anion radical to oxygen and H_2_O_2_, was up-regulated in aleurones treated with GA or C646 (Fig. 7A and B). These results indicated that GA induced the accumulation of H_2_O_2_ in aleurones by triggering the expression of H_2_O_2_ synthesis genes and suppressing the expression of H_2_O_2_ catabolism genes. To further understand the role of DNA methylation in maize aleurone PCD, we treated aleurones with RG108 in the presence of GA or C646. The expression of H_2_O_2_ production related genes was verified by qRT-PCR. Both C646 and GA treatment induced a reduction in *POD* gene expression levels and an increase in *SOD* gene expression levels, but addition of RG108 could prevent these changes (Fig. 7A and B). We further examined the content of H_2_O_2_, a significant increase in the content of H_2_O_2_ was observed after GA or C646 treatment; however, the methylation inhibitor RG108 could inhibit the production of H_2_O_2_. Thus, ROS signaling may be regulated by DNA methylation, playing a key role in GA-induced aleurone PCD. Furthermore, we performed sodium bisulfite genomic sequencing to investigate the methylation status of CpG islands in the *SOD* gene promoter after GA or C646 treatment as compared with untreatment. Genomic DNA was isolated from aleurone layers, and at least seven available cloning sequences were obtained from sequencing. The position of 23 CpG sites in a 300 bp *SOD* gene promoter region was obtained. Sodium bisulfite sequencing results showed that most CpG sites were significantly methylated after treatment with GA or C646 compared with the control (Fig. 8). Intriguingly, the expression of the *SOD* gene was not silenced even though the *SOD* promoter was hypermethylated under GA or C646 treatment. A decrease in the methylation level in the promoter region was observed at 23 CpG sites in GA-treated aleurone layers in the presence of RG108 (Fig. S5). This strengthened our conclusion that GA induces DNA methylation that regulates H_2_O_2_ production in aleurones.

## Discussion

**Fig. 9:**
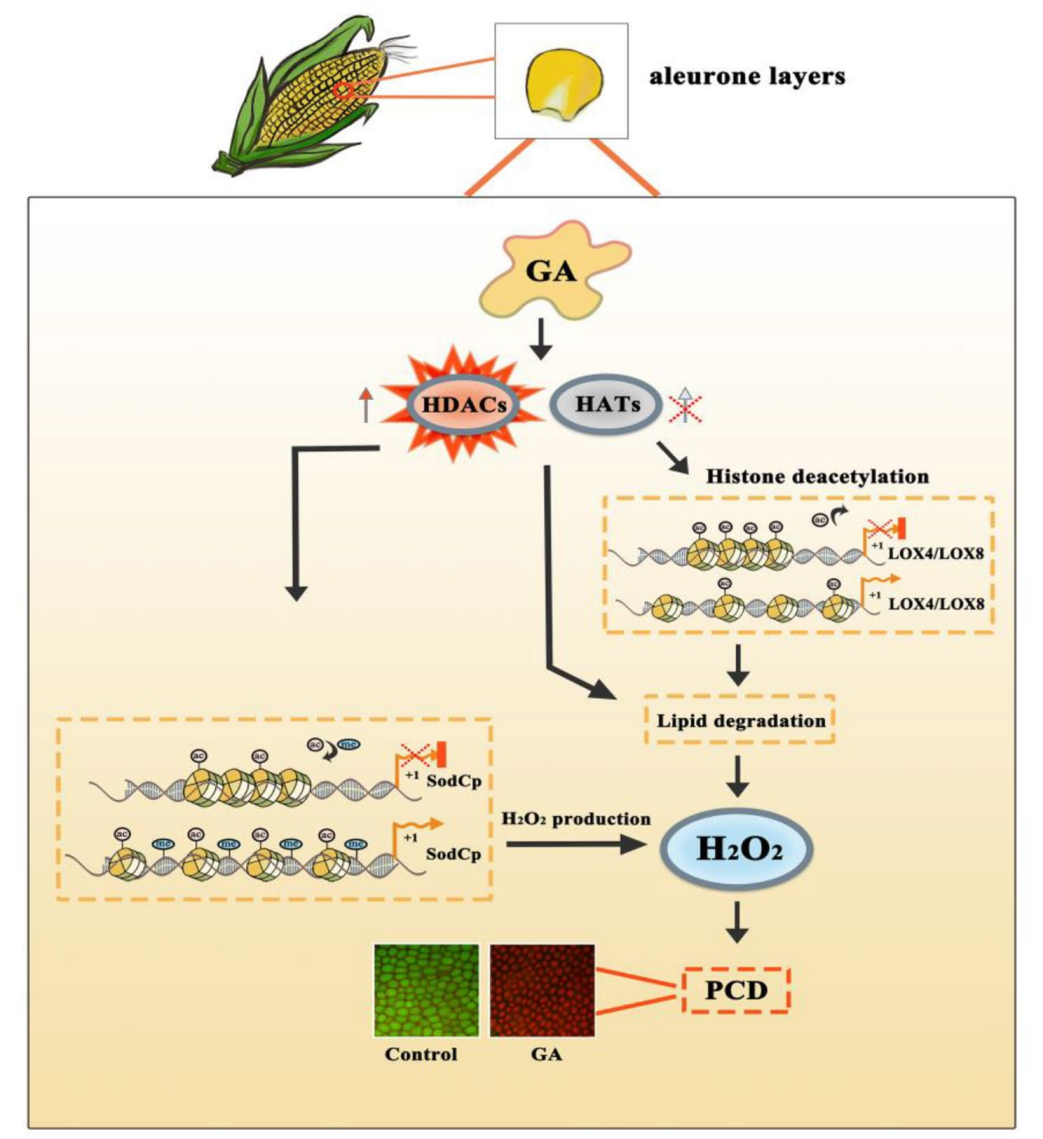
Model of regulation of GA-induced maize aleurone PCD through DNA methylation and lipid metabolism pathway. GA and HAT inhibition promote the degradation of lipid by elevating the activities of HDACs, and HDACs increase DNA methylation and modulate expression of H_2_O_2_ metabolism related genes, resulting in accumulation of H_2_O_2_ and cell death in maize aleurones

Aleurones start to enter PCD shortly after seed is germinated; however, the molecular mechanism underlying PCD is largely unknown. Based on our previous research that exogenous application of HAT inhibitors could induce PCD in dissected maize aleurones even in the absence of GA, demonstrating that HDACs were required for PCD [9], and to better understand which genes and pathways, especially epigenetic signaling, participate in PCD in maize aleurone layers, we applied GA and a HAT inhibitor C646 to treat maize embryoless aleurones respectively, and produced transcriptome data through RNA-seq. Transcriptome analysis showed that the expression of genes involved in lipid metabolism and DNA methylation pathways as well as ROS metabolism was significantly modulated in aleurone layers in the presence of GA or the HAT inhibitor. Further detailed studies indicated that GA induced lipid and ROS metabolism via enhancing HDAC activity and DNA methylation, leading to PCD in maize aleurone layers (Fig. 9).

### DNA methylation signaling pathways are involved in PCD in maize aleurones

Many observations suggest that both DNA methylation and deacetylation of core histones are frequently associated with the repression of gene expression [38–40]. Analysis of DNA methylation demonstrated that TSA could cause selective loss of methylation in Neurospora [41]. Some reports have suggested that histone H4 hyperacetylation affects DNA methylation levels [19, 42]. It is well established that DNA methylation can also lead to the recruitment of HDACs [21]. Our previous study indicated that histone deacetylation occurred during the PCD process of maize aleurone layers [9]. This study showed that DNA methylation increased during GA-promoted PCD in maize aleurones and the DNA methylation inhibitor RG108 or 5-AC could inhibit PCD induced by GA or the HAT inhibitor C646, indicating that C646 or GA induces PCD through increasing the DNA methylation level. These results suggested that GA upregulated HDACs, which in turn induced an increase in DNA methylation levels, leading to PCD of the maize aleurones. How histone deacetylation caused DNA methylation in aleurone cell death is still unclear.

GA is one of the most important factors promoting aleurone cell death, in which several components, including H_2_O_2_, GAMyb and PKABA, have been identified as major PCD regulators. These regulators function downstream of GA-dependent PCD pathways by directly and indirectly regulating the expression of some PCD-related genes [43]. Evidence has recently emerged that epigenetic modification is another key regulator of PCD [9, 44]. To better understand how GA signaling and epigenetic signaling regulation network regulates PCD of the aleurone layers, the present study showed that maize aleurones underwent a dramatic increase in DNA methylation during PCD in response to GA or HAT inhibitors. Hundreds of genes were up-regulated during GA-induced PCD in aleurone cells, accompanied with an increase in total DNA methylation levels, suggesting that DNA methylation plays an important role in regulating PCD. It remains unclear, however, how DNA demethylation contributes to PCD and PCD-associated changes in DNA methylation patterns. These results suggest that DNA methylation together with HDACs regulates the expression of PCD-related genes, leading to PCD in aleurones.

### GA-induced DNA methylation is associated with H_2_O_2_ generation via lipid breakdown

Aleurone cells have abundant glyoxysomes and stored lipid reserves, which are a significant source of ROS [35]. It has been proposed that lipoxygenase plays a key role in neutral lipid degradation [33]. Lipoxygenase has also been shown to produce ROS in plants. The GO term “glyoxylate metabolism” was further enriched following GA treatment, as described above. A variety of oxidases have the potential to generate ROS. A by-production of these reactions is the synthesis of H_2_O_2_ [45]. Thus we proposed that aleurones metabolized neutral lipids via the glyoxylate cycle and produced ROS in response to GA or the HAT inhibitor. Our transcriptome analysis showed that lipid hydrolysis was enhanced after subjected to GA, but further studies showed that lipid storage degradation was not affected when DNA methylation was inhibited. However, the *SOD* gene was down-regulated and the *POD* gene was upregulated, leading to no accumulation of H_2_O_2_ observed after treatment with the DNA methylation inhibitor. We previously showed that H_2_O_2_ regulated PCD in maize aleurones and ROS-related enzyme such as SOD and POD were regulated by HDACs [9]. It appears that DNA methylation also participates in regulation of expression of these enzyme genes during PCD. The results suggested that GA and C646 mediated the lipid metabolism process, and DNA methylation was involved in the production of H_2_O_2_ and PCD in aleurone layers.

### DNA methylation functions as an activation mark for the expression of many genes during PCD in maize aleurones

DNA methylation can alter the chromatin state by adding or removing methyl at cytosine on DNA, and is an important epigenetic mark participating in plant growth and development, and environmental responses through regulation of gene expression. Transcriptome analysis found that 345 DEGs were up-regulated and the remaining 567 DEGs were down-regulated after treated with GA or C646 for 3 d, accompanied with increased global DNA methylation levels. DNA methylation is always associated with silent transcription. A study in Arabidopsis DNA demethylase mutants showed that some genes were silenced due to hypermethylation [46]. In tomatoes, demethylase *sldml2* mutations caused DNA hypermethylation, leading to the silencing of hundreds of genes [15]. However, recent studies revealed that DNA methylation could also activate the expression of many genes. For example, it has been shown that transcriptional silencing occurs due to promoter DNA hypomethylation, and by contrast, promoter DNA hypermethylation enhances the gene expression level [47]. In tomatoes, many genes are repressed by DNA hypomethylation during ripening [15]. Similarly, the histone deacetylase (HDAC) is generally believed to repress gene transcription, but some reports showed that HDACs could also activate gene expression [48]. Our results showed that DNA methylation enhanced PCD-related gene transcription. We speculate that DNA methylation unsilences gene transcription through remodeling of chromatin, preventing the binding of certain transcriptional repressors or facilitating transcriptional activator recruitment [15, 49].

## Conclusions

Transcriptome analysis of maize aleurone cells treated with GA or HAT inhibitor C646 showed significant differences in the expression of genes related to DNA methylation, lipid metabolism, and ROS signaling. Our data suggest that GA-induced higher HDAC activity in aleurones causes breakdown of lipids via regulating lipoxygenase gene expression, and increased DNA methylation positively mediates H_2_O_2_ production. Therefore, DNA methylation and lipid metabolism pathways may play a role in maize aleurone PCD. Further studies are required in the future, including how GA regulates HDACs and which HDACs are involved in maize aleurone layer PCD.

## Materials and methods

### Plant materials

Seeds of the inbred maize (*Zea mays*) female parent Jingying8 were used in this study. As described by [9], embryoless aleurone layers were isolated from seeds of the maize. The HAT inhibitor C646 (purity, 99.66%) (Selleck, S7152) was dissolved in DMSO to concentration of 50 mM. Experimental group aleurone layers were treated with 100 µM GA (Biosharp, BS018) and 10 µM C646, respectively. And control group aleurone layers were treated with diluted DMSO. Aleurone layers were cultivated on sterilized 1/2 MS medium in an artificial climate chamber at 25^°^C, dark regime, and 70% humidity.

### Sample collection and RNA preparation

Experimental group and control group aleurone layers were sampled for three biological replicates after treatment for 1 d, 2 d, 3 d, 4 d and 5 d, respectively. Tissues of each group were mixed and total RNA was extracted using the RNA-prep pure Plant Kit (Qiagen, Mannheim, Germany) based on the manufacturer’s instructions. Agarose gel electrophoresis (Lonza, Switzerland) was used to confirm the integrity of all RNA samples, and a NanoDrop 2000c Spectrophotometer (Thermo Scientific, USA) was used to measure RNA concentration.

### Illumina library construction and RNA sequencing

A total of 45 RNA libraries were constructed from the treated maize aleurone layers for 1 d, 2 d, 3 d, 4 d and 5 d, respectively, for RNA sequencing. A total amount of 1 µg RNA per sample was used as input materials for library construction. The libraries were constructed and sequenced by Biomarker Biotechnology Corporation (Beijing, China). The NEB NextR UltraTM Directional RNA Library Prep Kit for IlluminaR (NEB, USA) was used to construct sequencing libraries, and they were purified with AMPure XP Beads (Beckman Coulter, Beverly, USA) for selective cDNA fragments of 240 bp in length. Next, the library quality was assessed on the Agilent Bioanalyzer 2100. Clustering of the samples was then performed on a cBot Cluster Generation System using TruSeq PE Cluster Kitv3-cBot-HS (Illumina) according to the manufacturer’s instructions. Finally, the library preparations were sequenced on the Illumina HiSeq Xten platform.

### Data analysis

The raw data from the Illumina HiSeq Xten platform were processed by using the Fastq software. In this step, aptamer-containing reads, ploy-N-containing reads, and low-quality reads are removed from the raw data to obtain clean data (clean reads). All downstream analyses are based on high quality clean data. These clean reads are then mapped to the reference B73 v4 genome sequence using the Hisat2 software tool. The alignment results of each sample were sorted and merged after duplicated reads were removed by picard tools v1.41 and samtools v0.1.18. The expression level of all genes was estimated by FPKM (Fragments Per Kilobase of transcript per million fragments Mapped). Differential expression of the two conditions/groups was analyzed using an edgeR software. The resulting p-values were corrected using the method of Benjamini and Hochberg to control for false discovery rates. Genes with edgeR corrected p-values < 0.05 were considered to be differentially expressed.

### Gene functional annotation

Nr (NCBI non-redundant protein sequences), Nt (NCBI non-redundant nucleotide sequences), Pfam (Protein family), KOG/COG (Clusters of Orthologous Groups of proteins), Swiss-Prot (A manually annotated and reviewed protein sequence database), KO (KEGG Ortholog database) and GO (Gene Ontology) databases were used to annotate gene function [50–52].

### qRT-PCR

For transcription analysis, qRT-PCR was performed to confirm the differential expression pattern of genes selected in the RNA-seq experiments according to the method previously described by [53]. Total RNA was isolated from the aleurone layers, complementary DNA was generated using HiScript® II Q Select RT SuperMix for qPCR (+ gDNA wiper) (Vazyme, China), and qRT-PCR was performed in 20 µl reactions using the SYBR Green Real-Time PCR Master Mix (Toyobo) on a StepOne Plus real-time PCR system (Applied Biosystems, USA). qRT-PCR primers are listed in Table 1. The results were repeated from three biologically independent experiments.

**Table 1:** Primers used for qRT-PCR

Primer	Sequence (5’-3’)
*Zm00001d006252*	AATTGAGCGTGAGATTGGCAG
	CCAGGTACATTGGTTTTGAGGA
*IDM1*	GCGGAGATGCATACCATGAC
	GACATGACTACGCAGCCCTA
*ROS1*	CACACAATCCAGAGCGAGGT
	TCCCCAACTGAGGCTCAAGA
*LOX4*	AGTCGAGTTTACGAGGTATGTTTT
	ATACCCCTACCGAAAACGCC
*LOX8*	GAGACCGACCCAAGAAAGGG TTCTTCTCGAACCAGCCCAC
*POD*	GTCATCGGCGGTCCGTTC AAGGACTGGAGGAGTTGGGT
*SOD*	CGTGCTACTCTGTCTCTGCT GAGACAAGAGTGGGAATGGCT

### Dot-blot analysis of DNA methylation levels

A dot-blot immunoassay of DNA methylation was performed on genomic DNA samples spotted on DEAE membranes to determine changes in overall DNA methylation levels in aleurone layers after different treatments according to the procedure described by [54]. DNA was spotted onto a DEAE membrane, and anti-5mC antibody was used to detect cytosine methylation in the different DNA samples. The mean gray values of the signal intensity were measured using Image J.

### Chromatin immunoprecipitation (ChIP)-PCR

ChIP-PCR of the promoter region (regions A-E) of lipoxygenase genes *LOX4* and *LOX8* were carried out using H3K9ac and H4K5ac antibodies following the procedure reported by [9]. The nucleus was isolated from aleurone layers by liquid nitrogen grinding. Chromatin was subjected to ultrasonic treatment, and then chromatin binding to protein A was incubated with H3K9ac and H4K5ac overnight at 4^°^C. Purified DNA from the ChIP samples was used for quantitative real-time PCR. The primer sets used in this test are listed in Table 2. A negative control was performed using rabbit serum for mock immunoprecipitation.

**Table 2:** Primers used for ChIP

Primer	Sequence (5’-3’)
*LOX4* Set-A	TTAACACGAGACGCACGACA
	CGACAGCGACGTGTATACGAA
*LOX4* Set-B	ACATTCATCAGGACGGCACG
	CCAGGTCCCTCTCCCTTCAG
*LOX4* Set-C	TAACGCAGAAGTCAGGTGCAA
	AGTGTTCCTGTCCTGTCCTG
*LOX4* Set-D	TGTTTGGTTCGTATATTTAGGGGA AGCTTGAATAGATCAGCATCACT
*LOX4* Set-E	TTCCATCTAAGGCGCATCCA AGCTAGAGAGGAGGAAGGACG
*LOX8* Set-A	AACCAAACAGCCCCTGAGTT GTTGCTGATGCGGACAACG
*LOX8* Set-B	TTCTGTGTAGCAGTGTAGGAGG TATGCGGAACAAACGACGCA
*LOX8* Set-C	GCCCTTATACGCGTCCCTC TGATGGCCGGTCCTTTTGTT
*LOX8* Set-D	GTCGAGGCCCACTTACTGC GCATGTGTTGTTTCCGCTTCT
*LOX8* Set-E	AGCAGATCAGTGCATGTGGG GAATGGACAGCCCTTTTGCC

### Sodium bisulfite modification

A total of 1–2 µg genomic DNAs were denatured and bisulfite converted by EZ DNA Methylation™ Kit (ZYMO Research, USA). Unmethylated cytosine was bisulfite converted to thymine while 5-methylcytosine was not converted by bisulfite and remained as a cytosine. Bisulfite-modified DNA was used for PCR, recovered, purified and cloned. Plasmid DNA from clones was used for sequencing. The primers were: 5’-GTTGATGTGTTTTAATGAAAATGTTG-3’ and 5’-TAAATAAAAATAAAAACTTCCTCTC-3’.

### Neutral lipid isolation and measurement

The content of neutral lipids was measured by the Triglycerides TG Enzymatic Kit (Applygen Technologies, China). Approximately 50 mg aleurone layer tissues were ground into powder in liquid nitrogen and incubated with pyrolysis solution for 10 min. The supernatant after centrifugation was collected and incubated at 70^°^C for 10 min. For the control and standard curves, standard buffer was added instead of lipid extract. For the blank, pyrolysis buffer was added into the blank wells. And then all samples were incubated at 37^°^C for 15 min. The absorbance could be read on a microplate reader at 550 nm.

### H_2_O_2_ measurements

The concentrations of H_2_O_2_ were measured according to the procedure described by [9]. Samples were extracted with acetone from aleurone layers and reacted with 5%(w/v) titanium sulfate to form a yellow precipitate, and then dissolved in H_2_SO_4_. Finally, the absorbance could be read on a microplate reader at 415 nm.

### Electronic supplementary material

Below is the link to the electronic supplementary material.

Supplementary Material 1

Supplementary Material 2

Supplementary Material 3

Supplementary Material 4

## Data Availability

All data supporting the conclusions described here are provided in tables, figures and additional files. The datasets during the study are deposited in Genome Sequence Archive (GSA) repository (Genome Sequence Archive - CNCB-NGDC), and the GSA accession number: is CRA012355.

## Abbreviations

HDAC: Histone deacetylases
HAT: Histone acetylase
DEGs: Differentially expressed genes
H_2_O_2_: Hydrogen peroxide
PCD: Programmed cell death
GA: Gibberellin
ROS: Reactive oxygen species
DMSO: Dimethyl sulfoxide
qRT-PCR: Quantitative real-time PCR
DEAE membrane: Diethylaminoethyl cellulose membrane
KEGG: Kyoto Encyclopedia of Genes and Genomes
POD: Peroxidase
SOD: Superoxide dismutase
ChIP: Chromatin immunoprecipitation
