# Supplementary material for: DNA methylation and lipid metabolism are involved in GA-induced maize aleurone layers PCD as revealed by transcriptome analysis
Source: BMC Plant Biol. 2023 Nov 22;23:584. doi: 10.1186/s12870-023-04565-5 (PMC10664605; doi:10.1186/s12870-023-04565-5)
Supplement: Supplementary file 1 — Supplementary Material 1 [file 12870_2023_4565_MOESM1_ESM.docx]

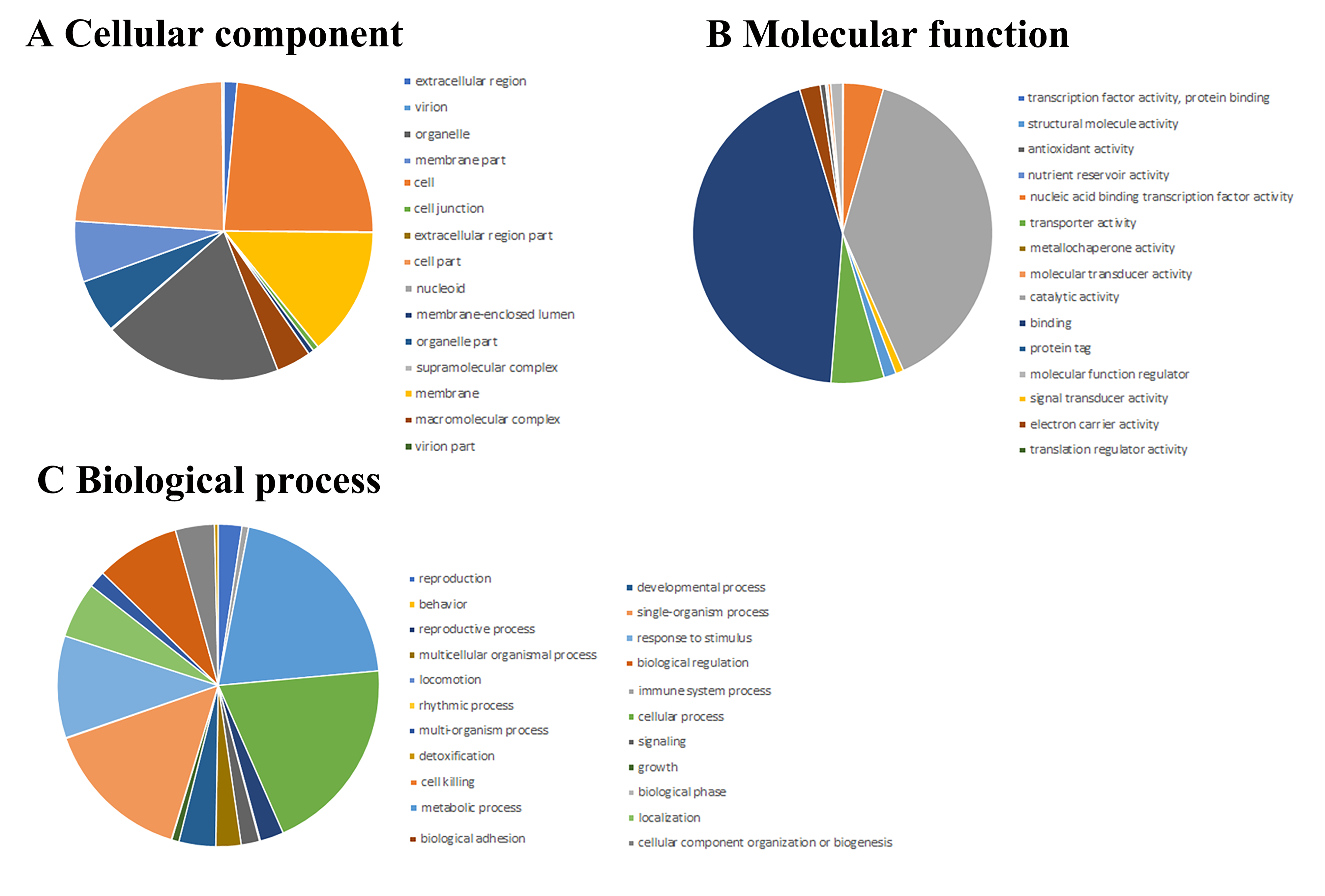


**Supplemental Figure S1.** The GO classification analysis of the 2554 DEGs. **A.** The DEGs participated in cellular components at the level 2. **B.** The DEGs showed molecular functions at the level 2. **C.** The DEGs were involved in biological processes at the level 2.


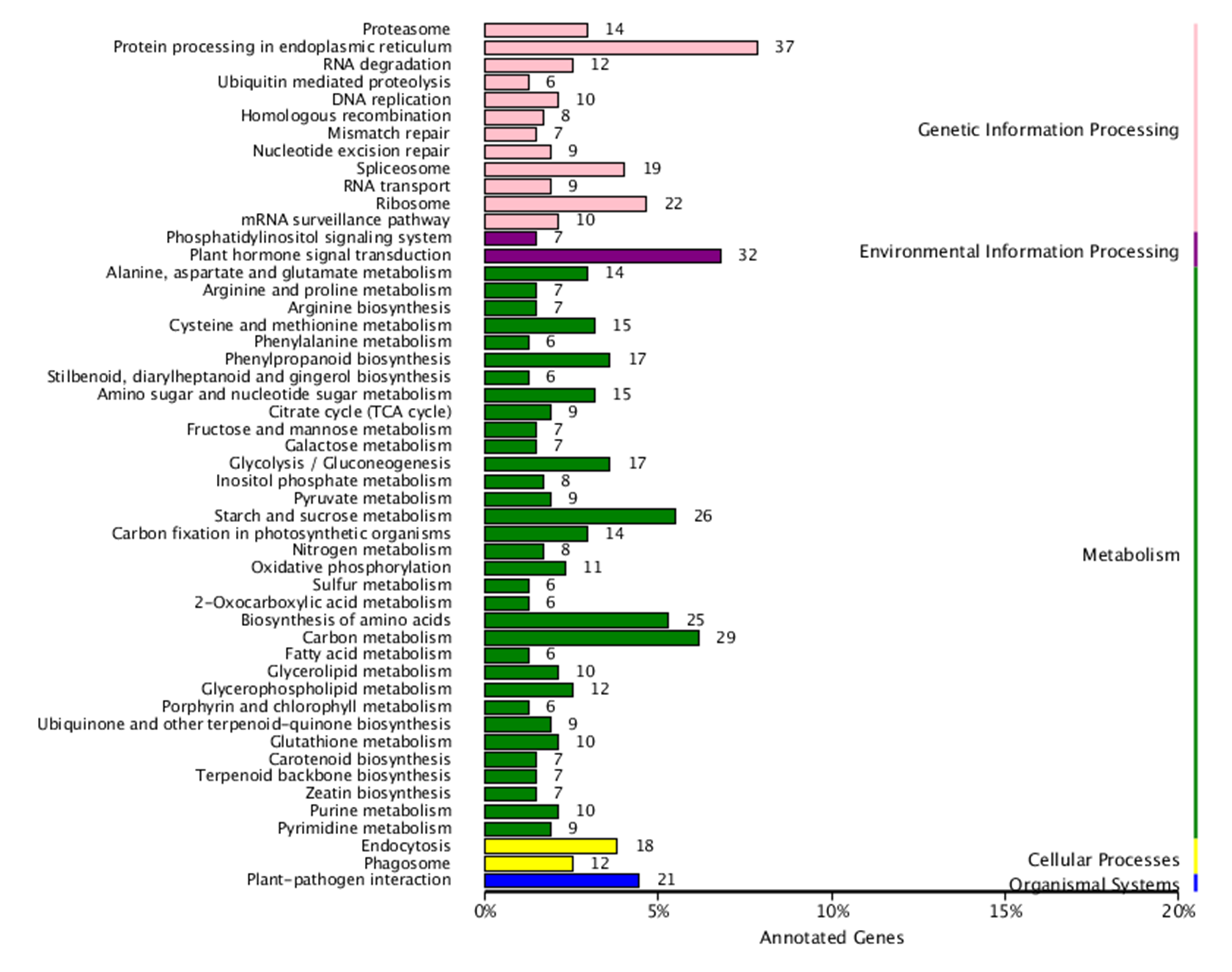


**Supplemental Figure S2.** The KEGG pathway analysis of 2554 DEGs. The different KEGG pathways of DEGs in aleurone layers after GA or C646 treatment.

**Supplemental Figure S3.** Analysis of effects of DNA methylation inhibitor 5-AC on GA or C646 mediated PCD in aleurones monitored by FDA/PI staining. Bar = 100 μm.


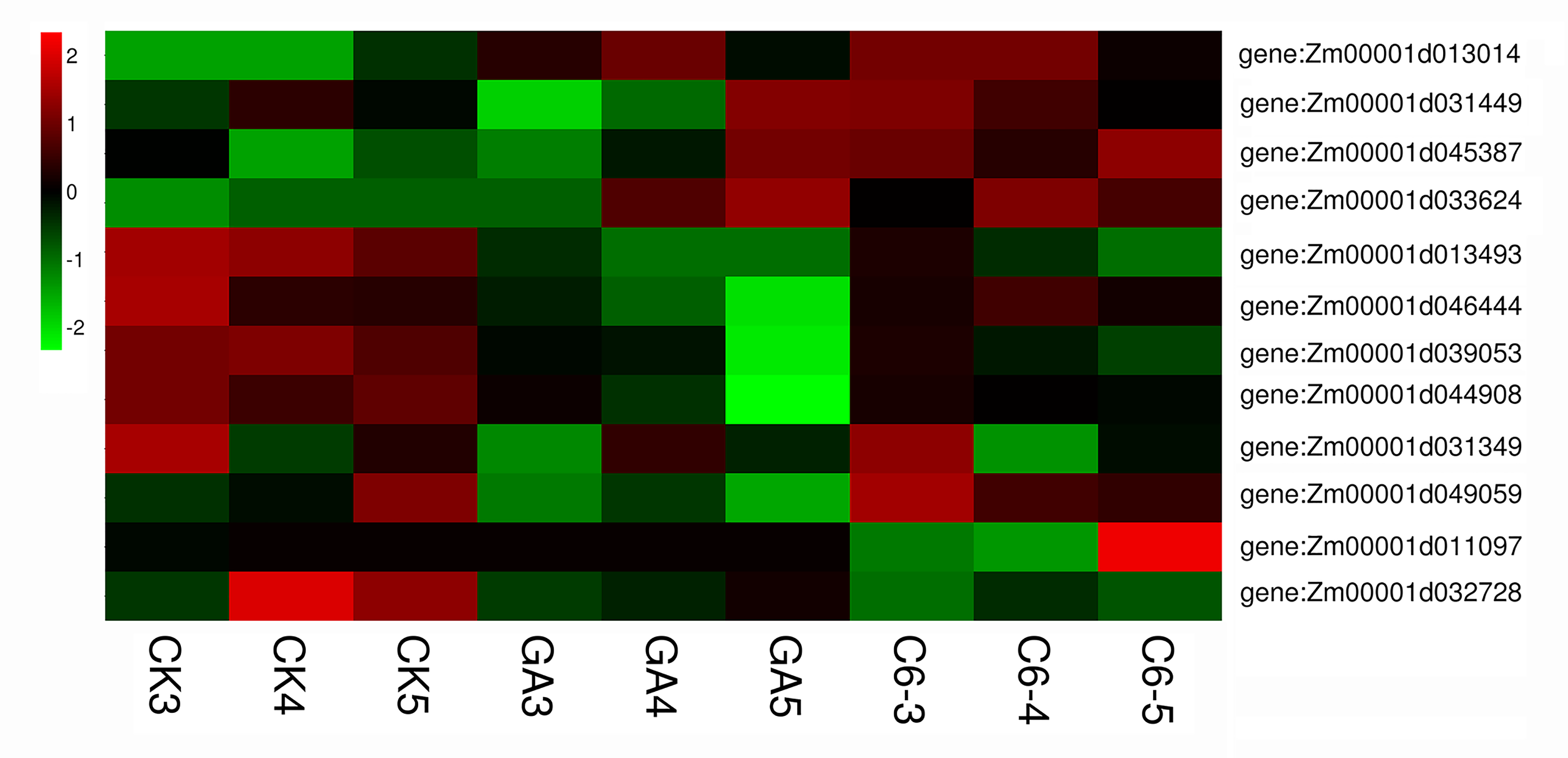


**Supplemental Figure S4.** The heatmap of genes involved in lipid metabolism.


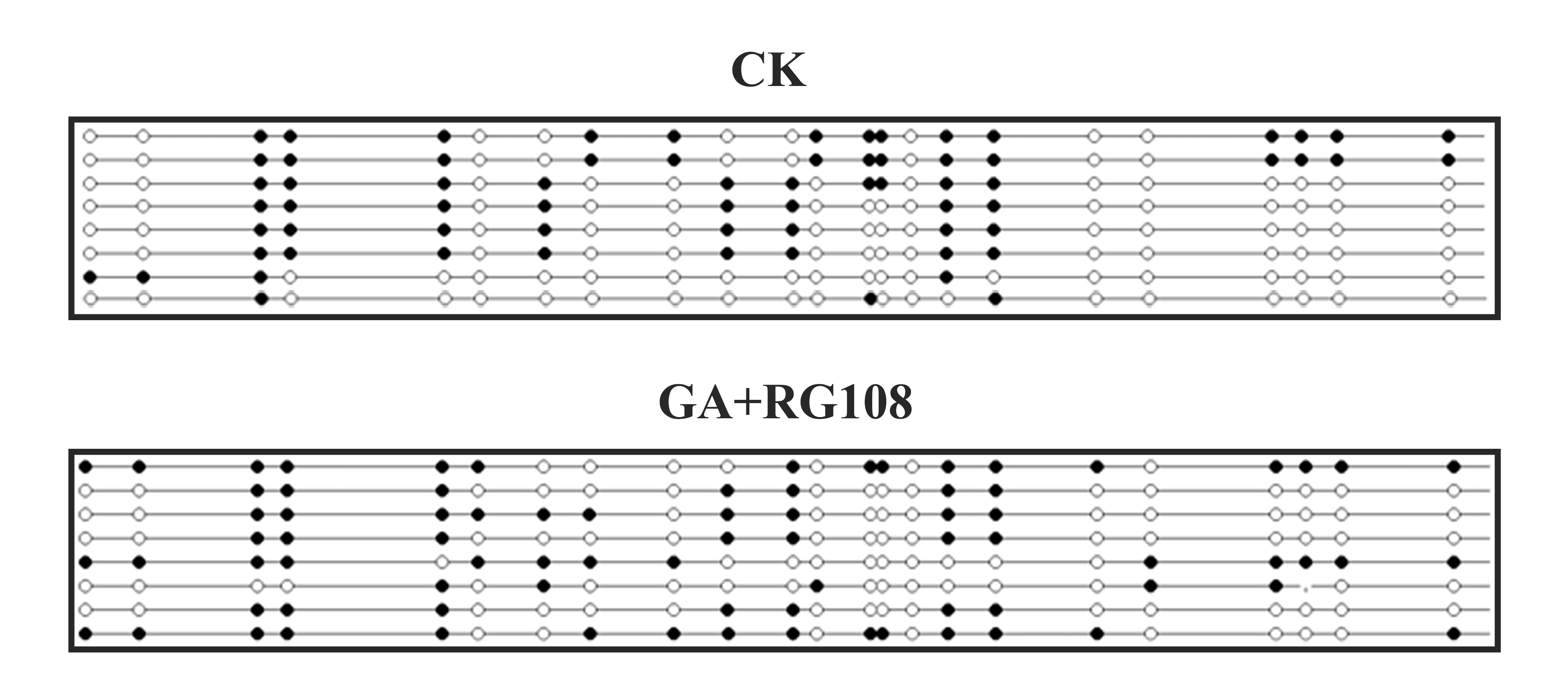


**Supplemental Figure S5** Effect of RG108 treatment on the methylation level of the *SOD* gene promoter. The filled circle represents unmethylated CpG dinucleotides and the open circle represents methylated CpG dinucleotides. RG108 inhibited an increase in the methylation level induced by GA.
